# Effect of feeding guar meal on nutrient utilization and growth performance in Mahbubnagar local kids

**DOI:** 10.14202/vetworld.2016.1043-1046

**Published:** 2016-10-04

**Authors:** Razia Sultana Janampet, Kishan Kumar Malavath, Rajanna Neeradi, Satyanarayana Chedurupalli, Raghunandan Thirunahari

**Affiliations:** 1 Department of Livestock Production Management, College of Veterinary Science, PVNR Telangana Veterinary University, Rajendranagar, Hyderabad, Telangana, India; 2 Department of Instructional Livestock Farm Complex, College of Veterinary Science, PVNR Telangana Veterinary University, Korutla, Telangana, India; 3 Department of Veterinary and Animal Husbandry Extension Education, College of Veterinary Science, PVNR Telangana Veterinary University, Rajendranagar, Hyderabad, Telangana, India; 4 Department of Instructional Livestock Farm Complex, College of Veterinary Science, PVNR Telangana Veterinary University, Rajendranagar, Hyderabad, Telangana, India

**Keywords:** growth performance, guar meal, nutrient utilization

## Abstract

**Aim:**

This study was conducted to evaluate the growth performance and nutrient digestibility of guar meal, an unconventional protein-rich feed ingredient in kids in comparison to conventional groundnut cake.

**Materials and Methods:**

A total of 18 kids were randomly allotted to three groups, and T1 group was fed on basal diet, T2 and T3 groups were offered diet replacing groundnut cake at 50% and 100% with guar meal, respectively, for a period of 120-day. At the end of the growth trial, a digestibility trial was conducted to evaluate the nutrient utilization.

**Results:**

There was no significant difference in dry matter intake among three groups. Nutrient digestibilities were significantly higher (p<0.05) in kids fed T2 ration with 50% replacement of groundnut cake with guar meal.

**Conclusion:**

It can be concluded that guar meal can be incorporated at 50% level in the concentrate mixture of goats replacing groundnut cake without any adverse effects.

## Introduction

Livestock is an integral part of agriculture and plays an important role in contributing to national economy. Although we have a large population of livestock, productivity is too low which is due to negligence in feeding regimen as the poor farmers cannot feed their animals adequately and the major constraint for this being the shrinkage of grass lands, erratic monsoons, and other human interventions with nature. India faces a net deficit (%) of 62.7 green fodder, 22.5 dry crop residues, and 64.0 concentrate feeds [1]. There is a serious shortage of conventional feed resources. Hence, it is necessary to look for protein rich, nonconventional feed resources. Guar meal is one such feed.

Guar (*Cyamopsis tetragonoloba*) is a drought-tolerant annual legume mostly grown in India and Pakistan [2]. Its cultivation in India is concentrated in North-Western states, namely Rajasthan, Haryana, and Gujarat. Gaur seeds are dicotyledonous consisting three parts: Endosperm, germ, and hull. Guar meal is obtained after mechanical separation of endosperm from both germ and hull of guar seed. It is a mixture of germ and hull at an approximate ratio of 1:3 [3]. Guar meal contains 40-50% protein [4]. Being a rich source of proteins, nutrients, and fiber with high digestibility, it is being used as feed after processing for livestock including fishery industry.

Guar meal is considered as a potential unconventional feed resource for livestock. The studies on the utility of processed guar meal as goat feed are limited. Hence, this study was planned to determine the growth performance and digestibility of different levels of guar meal in kids.

## Materials and Methods

### Ethical approval

The experiment was approved by Institutional Animal Ethics Committee.

### Site of study

The experiment was conducted at the Department of Instructional Livestock Farm Complex, College of Veterinary Science, Rajendranagar, Hyderabad, Telangana, India.

### Growth trial

A growth trial of 120 days was conducted in the year 2015 from the month of January to April on 18 Mahbubnagar local kids aged 3 months with an average body weight of 9.46±0.15 kg, divided into three equal groups of 6 animals each following completely randomized design. All the kids were dewormed and vaccinated. T1 group was fed concentrate mixture containing groundnut cake whereas in groups T2 and T3, groundnut cake was replaced with 50% and 100% guar meal (toasted at 110°C for 20 min), respectively, and Hybrid Napier (APBN1) was offered *ad libitum* to all the three groups as roughage. The ingredient composition of experimental diets fed to kids is given in Table-1. Feed was weighed and offered to the animals once a day at 8.00 am and the feed refusals of each animal were weighed and recorded daily. Clean water was made available throughout the experiment. Animals were weighed for 2 consecutive days at the beginning and at fortnight intervals during the experimental period.

**Table-1:** Ingredient composition (g/kg) of feed ration.

Ingredient	T1	T2	T3
Maize	33	32	34
Deoiled rice bran	21	19	18
Wheat bran	18	19	17
Groundnut cake	18	9	-
Guar meal (toasted)	-	9	18
Molasses	7	9	10
Mineral mixture	2	2	2
Salt	1	1	1
	100*^≠^	100*^≠^	100*^≠^

* APBN1 is supplemented *ad lib* to all groups;

≠ Vitablend was added at 30 g/100 kg ration. T1, T2, T3 refers to the three experimental rations. T1=Control diet with groundnut cake, T2=Groundnut cake replaced with guar meal at 50% level, T3=Groundnut cake replaced with guar meal at 100% level

### Digestibility trial

After the growth trial four animals from each group were selected and a 7-day digestibility trial was conducted to assess the nutrient utilization. During the collection period, animals were shifted to separate metabolic cages. Before starting the collection period, animals were acclimatized to metabolic cages for 5 days. Feces voided during 24 h were collected using fecal bags harnessed to the kids. Representative samples of feed offered, residue left, and feces voided were taken daily, dried, pooled, and stored for further analysis.

### Chemical analysis

Guar meal, Hybrid Napier (APBN1), and the three experimental rations were analyzed for chemical composition and fiber fractions as per Association of Analytical Communities [5] and Van Soest *et al*. [6] methods, respectively.

### Statistical analysis

Statistical analysis of the data was performed according to the procedures suggested by Snecodor and Cochran [7]. Least-square analysis of variance was used to test the significance of various treatments, and the difference between treatments means were tested for significance by Duncan’s new multiple range and F-test [8].

## Results and Discussion

### Chemical composition

The percentage of dry matter, organic matter, crude protein, ether extract, crude fiber, nitrogen free extract, total ash, neutral detergent fiber and acid detergent fiber values for guar meal were 93.56, 94.66, 49.52, 3.59, 4.46, 37.09, 5.35, 44.05 and 22.59, respectively, on dry matter basis. The chemical composition of guar meal, APBN1 and the experimental rations with different levels of guar meal fed to growing kids is presented in Table-2. The percentage of crude protein in toasted guar meal was 49.52 on dry matter basis which was comparatively higher than other conventional protein sources commonly used in preparation of concentrate mixture. Crude protein value for the conventional protein sources such as groundnut cake, cottonseed cake, mustard cake, and soybean meal is 43.59, 32.64, 36.03 and 47.35, respectively [9]. Content of crude protein determined in toasted guar meal in this study was in agreement with the findings reported by Tyagi *et al*. [10] and Nidhina and Muthukumar [11].

**Table-2:** Chemical composition of experimental rations (% DM basis)*, roughage and guar meal.

Chemical composition	T1	T2	T3	APBN1	Guar meal
Proximate composition					
DM	91.13	91.00	91.08	25.4	93.56
OM	90.31	92.53	93.02	87.22	94.66
CP	17.02	17.05	17.0	8.75	49.52
EE	2.22	2.48	3.14	1.52	3.59
CF	9.36	9.82	10.12	36.0	4.46
TA	9.69	7.47	6.98	12.78	5.35
NFE	61.61	63.18	62.76	40.95	37.09
Van Soest fiber fractions					
NDF	54.42	57.21	61.59	57.54	44.05
ADF	15.6	14.11	13.57	52.91	22.59

* DM basis=Dry matter basis, T1, T2, T3 refers to the three experimental rations. T1=Control diet with groundnut cake, T2=Groundnut cake replaced with guar meal at 50% level, T3=Groundnut cake replaced with guar meal at 100% level. DM=Dry matter, OM=Organic matter, CP=Crude protein, EE=Ether extract, CF=Crude fiber, TA=Total ash, NFE=Nitrogen free extract, NDF=Neutral detergent fiber, ADF=Acid detergent fiber

### Growth

The daily average dry matter intake (DMI) (kg/d) of three experimental diets during growth trial was 0.54±0.02, 0.58±0.02 and 0.53±0.02 (Table-3). No significant difference (p>0.05) was found in the DMI among three groups. Similar findings were reported by Goswami *et al*. [12] and Jongwe *et al*. [13], but the results disagreed with the findings of Makki [14] and Salehpour and Qazvinian [15] who reported decreasing DMI with increasing guar meal percentage which might be due to difference in the percentage of inclusion rate of guar meal by individuals. Insignificant difference in DMI in the present can be attributed to the usage of toasted guar meal as processing removes beany odor and residual gum making it more palatable and another factor may be the addition of molasses which improves the palatability of feed and fodder.

**Table-3:** Body weight gain (kg) and ADG (g) of kids fed with experimental rations.

Experimental group	Body weight (kg)		Total weight gain (kg)	ADG (g)

	Initial	Final		
T1	9.38±0.18	14.82±0.22	5.43±0.08	45.28±0.56^a^
T2	9.55±0.33	15.22±0.34	5.67±0.08	47.22±1.15^a^
T3	9.45±031	14.52±0.35	5.07±0.08	42.22±0.88^b^

ADG: Average daily gain.

ab Means with different superscripts column wise differ significantly (p<0.01). T1, T2, T3 refers to the three experimental rations. T1=Control diet with groundnut cake, T2=Groundnut cake replaced with guar meal at 50% level, T3=Groundnut cake replaced with guar meal at 100% level

There was significantly (p<0.05) higher average daily gain in kids fed on ration T2 than on kids fed on T3, but the values were comparable with the control group. This might be due to significantly higher crude protein digestibility in kids fed on T2 ration. Similar findings were reported by Goswami *et al*. [12] in calves fed concentrate replacing groundnut cake with guar meal at 50% and 75% level but Sharif *et al*. [16] did not observe significant difference in weight gain in Sahiwal calves on replacing cottonseed cake with guar meal at 7.5% and 15% level in concentrate mixture.

### Digestibility and plane of nutrition

The DM intake (kg/day) during the digestibility trial was 0.53±0.02, 0.59±0.03 and 0.53±0.03, respectively, for T1, T2 and T3 and the values did not differ significantly (p>0.05). Digestibility coefficients (%) (Table-4) for dry matter, organic matter, crude protein, neutral detergent fiber and acid detergent fiber, crude fiber, and nitrogen free extract were significantly (p<0.05) higher for kids fed T2 ration but the digestibilities of T3 ration were comparable with T1 diet. No significant difference was found in the digestibility coefficient (%) of ether extract among three groups. Results of this study were in contrast to those reported by Goswami *et al*. [12] and Grewal *et al*. [17] who reported non-significant difference in digestibilities in calves and buffaloes, respectively. The digestible crude protein (DCP) intake (g/day) of kids fed T2 ration was significantly (p<0.05) higher than kids fed on T1 and T3 which was reflected in higher weight gains in T2 compared to other two groups. Total digestible nutrient (TDN) intake (g/day) and metabolic energy intake (MEI) per day (Mcal) were also higher in T2 group. All the groups met the requirement for DM, DCP, TDN and MEI recommended by ICAR [18].

**Table-4:** Effect of feeding different levels of guar meal on dry matter intake, nutrient digestibility and plane of nutrition in kids.

Parameter	Experimental ration		

	T1	T2	T3
Body weight	15.00±0.23	15.10±0.52	14.68±0.45
DMI (kg)	0.53±0.02	0.59±0.03	0.53±0.03
DM digestibility	62.56±1.15^b^	68.51±1.06^a^	60.23±1.44^b^
OM digestibility	67.87±0.96^b^	72.62±0.87^a^	65.00±1.19^b^
CP digestibility	66.53±0.94^b^	72.79±0.78^a^	63.03±1.59^b^
EE digestibility	75.02±0.65^NS^	77.53±0.65^NS^	77.37±0.65^NS^
CF digestibility	59.63±1.08^ab^	65.69±1.07^a^	52.96±3.60^b^
NFE digestibility	75.42±0.93^b^	77.72±0.86^a^	74.08±0.73^b^
NDF digestibility	54.92±2.16^b^	64.54±0.98^a^	55.52±1.36^b^
ADF digestibility	51.62±2.26^b^	59.62±1.05^a^	45.55±2.75^b^
DCPI (g/day)	59.99±0.42^b^	73.22±0.31^a^	56.76±0.48^b^
TDNI (g/day)	355.74±1.20^b^	426.45±1.15^a^	360.56±1.21^b^
MEI (Mcal/day)	1.31±0.11^ab^	1.55±0.16^a^	1.27±0.19^b^

ab Values bearing different superscripts in a row differ significantly (p<0.05), T1, T2, T3 refers to the three experimental rations. T1=Control diet with groundnut cake, T2=Groundnut cake replaced with guar meal at 50% level, T3=Groundnut cake replaced with guar meal at 100% level. DCPI=Digestible crude protein intake, TDNI=Total digestible nutrients intake, MEI=Metabolic energy intake, DMI=dry matter intake, ADF=Acid detergent fiber, DM=Dry matter, OM=Organic matter, CP=Crude protein, EE=Ether extract, CF=Crude fiber, TA=Total ash, NFE=Nitrogen free extract, NDF=Neutral detergent fiber, NS=Not significant

## Conclusion

Results of this study indicated that replacement of 50% groundnut cake with guar meal improved the growth performance and nutrient digestibilities without any adverse effects on experimental kids. Hence, it can be incorporated in the ration in place of conventional feed ingredients.

## Authors’ Contributions

KKM, RN, SC and RT planned, guided and supervised the entire research work. RSJ carried out the experimental work, laboratory analysis, and data analysis. Manuscript preparation was done by RSJ under the guidance of RN. All authors read and approved the final manuscript.
